# Influence of Social Media Uses and Gratifications on Family Health among U.S. Parents: A Cross-Sectional Study

**DOI:** 10.3390/ijerph20031910

**Published:** 2023-01-20

**Authors:** Eliza Olpin, Carl L. Hanson, AliceAnn Crandall

**Affiliations:** Department of Public Health, Brigham Young University, Provo, UT 84602, USA

**Keywords:** family health, social media, uses and gratifications, connection, entertainment

## Abstract

Some research suggests that parents on social media have access to greater social support and health information. However, evidence also connects parental social media use to negative outcomes including increased parental stress, depression, and distraction. Using the uses and gratification theory, this study goes beyond measures of parents’ individual mental health and explores social media use and its association with family well-being. Family health outcomes were predicted to vary with parents’ use and gratifications of social media, with parents who use social media primarily for information and connection scoring higher on family health and parents who used social media for entertainment scoring worse on family health. The sample included 482 heterosexual married or cohabiting partners recruited through a Qualtrics panel. All participants were parents of children ages of 3–13, with mothers and fathers each completing the survey. Findings indicated that fathers’ use of social media for entertainment and connecting with family and friends was associated with better family well-being and health resources (*p* < 0.01). However, mothers’ use of social media did not have a statistically meaningful relationship with family health variables. There was no evidence that parental social media use was associated with negative family health outcomes. Longitudinal data is needed to determine the temporal relationship between social media use and family health. Public health professionals interested in improving family health may consider how to better reach fathers on social media to increase health resources.

## 1. Introduction

Social media applications are widely used by parents and children and have become part of family life. They bring new communication and information access opportunities for families related to parenting, health, and lifestyle [1,2,3]. Yet, many parents experience challenges as they find themselves overwhelmed by notifications, disheartened by social comparisons, or distracted by excessive consumption of social media content [4,5,6]. Concerns over social media use (SMU) and its effect on individual well-being and family functioning have received much attention in mainstream media [7,8,9]. However, few SMU studies exist that look beyond individual family members’ mental health to the larger family system. As such, researchers have yet to fully understand the relationship between social media use and family well-being—specifically, parental motivations for SMU and how those motivations may relate to aspects of family health. Insights from this study should encourage professionals to re-examine how they use digital tools to reach parents and promote health, and provides confidence regarding the assessment of SMU on family health outcomes.

### 1.1. Uses and Gratification Theory

The uses and gratifications theory is commonly used to explain motivations for SMU including how individuals choose media to satisfy specific needs or desires. According to Pelletier and colleagues, “by understanding the intrinsic needs that draw consumers toward specific media, Uses and Gratification Theory further helps recognize consumers’ motives for usage behaviors to receive gratification” ([10] p. 272). In the context of social media, this could include why people join one platform and not another, or what activities they engage in while using an application. 

The literature surrounding parents’ uses and gratifications of social media is limited, with social media constantly evolving. However, previous studies have found that parents are avid users of Facebook, with mothers typically more active on Facebook and other sites than fathers [11,12]. Several possible reasons, or gratifications, for parents’ SMU also emerge in the literature, namely: communication, connecting with friends and family, information, documenting family milestones, and entertainment [11,13,14,15]. Some of these gratifications overlap with the four major communication needs identified in Duffy and Thorson’s (2009) health communication media choice model: (1) connectivity, (2) information, (3) entertainment, and (4) shopping [16]. Connectivity refers to the need to relate, support, and engage with/communicate with others. Information refers to surveillance or the need to gain knowledge that is important for accomplishing goals. Entertainment refers to the need to be amused, relaxed, and diverted [16]. This model demonstrates how uses and gratifications of social media use intersect with health promotion.

Essentially, the uses and gratification theory help to explain the reason why social media may be used. In this paper, we explore whether those reasons were associated with better family health outcomes. For example, is using social media for obtaining information more highly associated with family health outcomes than using social media for connecting with others? As such, uses and gratification theory may help to better explain the potential specific social media antecedents to various family health domain areas. For purposes of the current study, shopping, or the need for acquiring goods and services, was excluded from the analysis because not all social media applications included had a shopping feature. 

### 1.2. Parental Social Media Use

Studies regarding parental SMU tend to focus primarily on two motivations: social support and information-seeking. Research points to SMU as a source of social capital and support [2,17,18,19]. For instance, in a study of 157 new mothers, more than 86% of mothers reported that they blogged to stay in touch with family and friends. Results of the study indicated that the frequency of blogging was positively associated with feelings of connection and social support [20]. 

Social media use for information-seeking by individuals may influence health outcomes [21,22,23]. As such, studies have explored parental SMU for seeking health information [24,25,26,27,28,29,30]. A systematic review of 12 studies revealed that parents of young children are largely motivated to use social media to obtain health information [31].

Additionally, SMU in general among parents is related to other personal outcomes such as feelings of anxiety, parental stress, depression from social comparison, and poor job performance [4,11,32,33]. A study of SMU among parents and their children during the COVID-19 pandemic found that high levels of anxiety were correlated with the likelihood to have used social media applications for both social support and information-seeking [34].

### 1.3. Social Media Use and Family Health

Although several previous studies of parents have searched for a relationship between SMU and external social supports, very few have sought to determine if SMU influences other aspects of family health [11,14,35]. Family health is a growing discipline in the field of public health and is defined as “the intersection of the health of each family member, their interactions and capacities, as well as the family’s physical, social, emotional, economic, and medical resources” ([36] p. 264). As a means of family communication, a source of social capital, or a method of health education, social media has the potential to influence family health in a variety of ways. These influences can be explored in relation to the four primary domains of family health: family social and emotional health processes, family healthy lifestyle, family health resources, and family external social supports [37]. Family social and emotional health processes include feelings of belonging, communication, and satisfaction in family relationships. Family healthy lifestyle includes healthy lifestyle habits, routines as a family, and the support family members give each other to engage in healthy activities. Family health resources include both internal and external health resources such as the health of family members, trust in medical professionals, knowledge of outside resources, health insurance coverage, access to transportation, adequate housing, and so forth. Family external social supports include individuals, groups, and organizations that the family can turn to when added support outside of the family is required to address needs [37].

Kerr and Bowen’s family systems theory posits that individuals in the family are interconnected and interdependent [38]. Although social media feeds are curated by individuals, one person’s online interactions may have an impact at the family level. For instance, studies have found a relationship between media use and marital quality [39,40]. Other research attests that the introduction of social media has changed communication patterns among families [15,41].

### 1.4. Aims and Hypotheses

**Figure 1:**
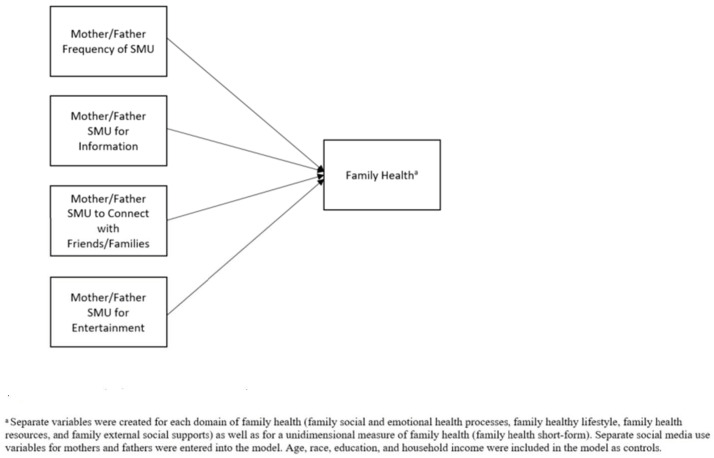
Conceptual Framework for the Relationship between Mother and Father Socail Media Use and Famliy Health.

Parents commonly use social media, but it is unclear to what extent social media use is associated with the health of the family. The purpose of this study was to determine if uses and gratifications for parental SMU were associated with family health outcomes. The study compared family health data as well as parents’ self-reported SMU in order to answer the following research questions: (1) Are some parents’ gratifications of SMU related to worse family health outcomes? (2) Is parents’ SMU positively associated with any family health outcomes? Figure 1 contains the conceptual framework for the study. The research questions previously mentioned resulted in the following two hypotheses:

**Hypothesis 1.**  *Family health outcomes would vary depending on SMU gratifications, with parents who use social media applications primarily for entertainment scoring worse on family health.*

**Hypothesis 2.**  *Parents who use social media primarily for information and connection would have higher family health.*

As it relates to the first research question, we expected that some parent gratifications of SMU, especially entertainment, would correspond with worse family health outcomes. This is due to recent literature suggesting that passive use of social media is associated with worse outcomes among adults [42,43]. Conversely, finding and sharing inspiring content on social media has been found to be associated with feelings of connection and a decrease in depressive symptoms [44].

Secondly, it was hypothesized that parents who use social media primarily for information and connection would have higher family health. Specifically, using social media for information and connection would help families increase their family health resources and external social supports subscales. Previous research has shown that, in some cases, social media has been linked to improved communication between family members and access to supportive networks [14,35,41]. These networks may be associated with better health outcomes for families. Social media platforms have become an integrated part of today’s society. As such, it is important to help parents understand the real risks and benefits of their participation and, perhaps even more importantly, how to use social media as a tool to promote the well-being of their family.

## 2. Materials and Methods

The sample comprised 482 heterosexual married or cohabiting partners (dyads), living in the United States, who were parents of children ages of 3–13 years. A Qualtrics panel was used to recruit couples and collect survey responses. A quota-sampling approach was used to ensure that there was racial and economic diversity. In particular, we required that a subset of the panel was from a racial minority group (at least one member of the dyad was non-white) and/or low education (at least one member of the dyad had less than a high school education). Qualtrics recruits participants through a series of panels that have agreed to participate in Qualtrics research. Only participants who met the eligibility requirements (e.g., married/cohabitating with a partner willing to participate, parents of a child 3–13 years of age) were able to complete the survey. Each dyad completed one survey through Qualtrics, with each partner responding individually to questions about their social media use and family health. Participants received compensation in the form of Qualtrics credit. The study was approved by the [blinded for review] Institutional Review Board.

### 2.1. Measures

**Family health.** Family health was measured using the 32-item Family Health Scale [37]. Both partners responded to the items. Response options were on a 5-point Likert Scale, ranging from *Strongly Disagree* to *Strongly Agree*. Higher scores indicated better family health. The Family Health Scale includes 4 subscales: family social and emotional health processes, family healthy lifestyle, family health resources, and family external social supports. Because responses from both members of a couple have been found to be similar and a unidimensional measure comprised of responses from both partners has been found to have good reliability and validity [45], average scores comprised of the responses of both partners were calculated for the 4 subscales. Additionally, 10 of the items (with items coming from all 4 subscales) made up an overall, previously validated family health short-form measure [45]. A family health short-form score was computed by averaging responses across the 10 items from both partners. Reliability for each of the subscales and the short form was good, with Cronbach’s alphas ranging from 0.88 to 0.92.

**Social media use.** For the purposes of this study, social media included apps and online networking sites that allow consumers to view, create, and share content online, curate a feed, and interact with other users. Social media use was examined by asking participants the frequency of their use of various social media platforms (Twitter, Instagram, Facebook, Snapchat, YouTube, WhatsApp, Pinterest, LinkedIn, and Reddit) on a 6-point Likert scale (never or almost never, every few weeks, 1 to 2 times a week, 3 to 5 times a week, once a day, or several times a day). An SMU frequency score was computed for each partner by averaging their responses across the 9 applications. Follow-up questions then identified participants’ primary purpose for using any of the applications they had reported using. Consistent with uses and gratification theory, response options included entertainment, relaxation, connecting with family/friends, documenting family milestones/activity, information, self-promotion, work, other, or did not use in the past 2 weeks. Separate scores for each partner were computed to understand if the participant used any social media applications primarily for information, connection with friends and family, and entertainment. 

### 2.2. Data Analysis 

Data were analyzed in Stata 17. Separate multiple regression models were run for each of the 4 family health subscales and the family health short form using the Stata *regress* command. Family health was regressed on social media frequency for each partner, and each partner’s use of social media for information, connection with friends/family, and entertainment. Each model controlled for mother’s age in years, mother’s race, mother’s education, and household income. Father’s age, race, and education were not included to avoid over-controlling due to the high correlation in age, race, and education between members of the dyad.

## 3. Results

### 3.1. Descriptive Statistics

The majority (90%) of couples were married, and most participants were white (74%). Mothers and fathers had similar levels of education, with about 68% of dyads having at least one member with a bachelor’s degree or higher. Almost half of the couples (46%) had an income of at least $100,000. Table 1 includes the demographic details of the sample.

### 3.2. Social Media Use

The top four most frequently used social media applications were Facebook, YouTube, Instagram, and Twitter (see Table 2). On average, parents reported using Facebook and YouTube daily, while Instagram and Twitter were used three to five days a week. Overall, parents’ SMU scores were similar; however, mothers reported SMU slightly more frequently than fathers in six of the nine applications. 

Almost all mothers (94%) and fathers (92%) reported having at least one social media application that they primarily used for entertainment (see Table 3). Likewise, a high proportion of mothers (79%) and fathers (71%) could identify at least one social media application that they used primarily for connecting with friends and family. More than half of participants (63% mothers, 60% fathers) reported using at least one social media application primarily for information purposes. 

### 3.3. Social Media Use and Family Health

Mothers’ SMU had little to no correlation with family health (see Table 4), although mothers’ frequency of SMU was associated with a 0.01 (*p* < 0.01) increase in family healthy lifestyle score. On the other hand, fathers’ use of social media to connect with family and friends was associated with a 0.19 (*p* < 0.01) increase in overall family health and a 0.37 (*p* < 0.01) increase in family health resources. Additionally, fathers’ use of social media for entertainment was also positively associated with overall family health (0.25, *p* < 0.05). Parental SMU was not associated with changes in family social and emotional health processes or family external social supports. Race and education were not associated with family health in any of the models. Participants who were older reported higher overall family health (*b* = 0.01, *p* < 0.01), higher family healthy lifestyle (*b* = 0.01, *p* < 0.05), and more family health resources (*b* = 0.02, *p* < 0.01). Household income was associated with better overall family health (*b* = 0.05, *p* < 0.001), family healthy lifestyle (*b* = 0.04, *p* < 0.01), more family health resources (*b* = 0.05, *p* < 0.05), and more external social supports (*b* = 0.05, *p* < 0.05).

## 4. Discussion

The primary purpose of this study was to determine if uses and gratifications for parental SMU were associated with family health outcomes. While several studies of parents have sought to understand the relationship between SMU and external social support [11,14,35], less is known about other motivations for SMU and their influence on family health. Uses and gratification theory provided the theoretical framework through which motivations for SMU were explored and explains how individuals select media to satisfy needs and desires [16]. Depending on the motivation for using social media, its potential influence on family health was explored across four family health domain areas including family social and emotional health processes, family healthy lifestyle, family health resources, and family external social support [37]. Results revealed slight variations in family health scores depending on the gratification for SMU. However, only fathers’ SMU had any statistically meaningful relationship with family health. Specifically, fathers’ uses of social media for connection and entertainment were associated with higher scores on family health short form and health resources scales. Mothers’ use of social media for information, connection, or entertainment showed very minimal change in family health measures. Although mothers’ SMU frequency was associated with higher scores on family healthy lifestyle, it was not statistically meaningful. 

### 4.1. Hypothesis 1: Parents Who Use Social Media Applications Primarily for Entertainment Would Score Worse on Family Health

According to the first hypothesis, parents’ use of social media for entertainment would be negatively associated with family health outcomes. There was no evidence, however, that entertainment or any other parents’ gratification of SMU was associated with worse family outcomes. This finding contradicted the negative effects associated with parental social media use identified in several studies [4,5,6,35,46]. One possible explanation for this result is that the term “entertainment” was not clearly defined in the current study and may include too wide a range of behaviors. For example, while some parents may view videos when bored, others may comment on a friend’s post. These differences could lead to ambiguity in the results. Additionally, the relationship between parental SMU and negative outcomes could be mediated by variables such as social comparison or problematic phone usage that were outside of the scope of this study [4,32,47]. Further research is needed to better understand how specific activities on social media, including those related to entertainment, may harm or benefit parents. 

### 4.2. Hypothesis 2: Parents Who Use Social Media Primarily for Information and Connection Would Have Higher Family Health

In the second hypothesis, it was predicted that parents who used social media for information and connection would report better family health. Yet, there was no evidence that fathers’ or mothers’ use of social media applications, primarily for information, was positively associated with any of the family health domains. A recent systematic review of parents’ health information-seeking behaviors acknowledged that parents’ behaviors on social media are understudied [29]. Some parents may have difficulty understanding health information found on social media due to conflicting opinions, discrepancies, and user confusion [26]. Research also shows that parents are often hesitant to trust health information found on social media platforms and other online sources [1,48,49]. Any of these factors could make it difficult for parents to apply health information in a practical way that would noticeably correspond to family well-being. 

In contrast, findings from the current study indicated that fathers’ use of social media to connect with family and friends was associated with a higher score on overall family health and family health resources. This relationship may possibly be explained through the entertainment function that SMU provides [50]. Current literature regarding fathers’ SMU and family well-being is limited, and studies have focused on the social support networks available to fathers online [51,52]. Interestingly, the current study did not find that fathers’ use of social media to connect with family and friends was associated with higher external family social supports. Yet, social media may still help fathers build connections that increase material resources, self-efficacy, and coping [11]. For example, one study noted that a Facebook group became a resource for black fathers looking for advice on finances, family expansion, relationship conflict, and child development [51]. Additionally, during the 2019 measles outbreak, parents’ communications on social media were found to increase self-efficacy and encouraged health prevention behaviors among U.S. parents [53]. It is important to note that without longitudinal data, it is impossible to determine the direction of the association between parent connection and family health resources. Fathers with more resources may also have more time and energy to engage with others on social media platforms. 

Overall, results indicate a need for additional research on how fathers interact with social media platforms. Future studies may seek to better understand how social media use influences fathers’ internal and external health resources available to the family. Likewise, researchers may also choose to study the activities that fathers engage in on social media when they wish to be entertained and how these correspond with an increase in their own and their family’s well-being. Although social media is undoubtedly complex, the current study showed no negative associations of SMU with family health, indicating that families are not necessarily suffering from parents’ SMU. These findings are notable because they counter a popular opinion that social media is harming society. Rather, with additional understanding, social media may be harnessed as a tool to strengthen family systems and improve well-being. 

## 5. Limitations

Findings from this study should be considered in the context of the following limitations. First, due to the study’s cross-sectional design, we cannot draw causal conclusions. Longitudinal data is needed to further examine directionality. Second, although we attempted to include a racially and economically diverse sample, parents with less than a high school diploma were underrepresented in this study and same-sex couples were not represented. As such, the results of the current study may not be representative of some populations. We did control for race, age, income, and education. However, a more diverse sample is needed to more fully investigate differences that may arise based on demographic differences that have been found in other studies [54]. Third, self-reported social media estimates have been found to have low accuracy and convergent validity [55,56,57]. Future studies may use more observable methods to measure the frequency of SMU. However, parents’ self-reported SMU is a valuable first step. 

Another consideration is that there may have been other uses and gratifications for SMU that were not examined in this study. For example, shopping, one of the major communications needs described by the health communication media choice model [58], was not included as a response option in questions asking about the parent’s primary use of each social media application. Finally, the most frequently used applications (i.e., Facebook, YouTube, Instagram, etc.) are often multipurpose tools. Many social media applications may not be “primarily” used for information purposes, yet parents still encounter and use health information from these sources. Qualitative data would be useful to better understand primary and secondary uses and gratifications of social media and its effect on family health and family context.

## 6. Conclusions and Implications

Findings from the study indicated that fathers’ SMU for entertainment and for connecting with family and friends was positively correlated with family well-being and health resources, while mothers’ SMU was not associated with family health. There was no evidence that parental SMU was associated with negative family health outcomes.

The results of this study suggest that social media may have additional uses in the field of health promotion as it relates to family health. First, health educators and program planners could consider how they might reach families by engaging more fathers through social media. For instance, they might choose to advertise health programming on apps that fathers frequent for entertainment. Secondly, community health and social workers could utilize social media as a tool to build clients’ connections and the number of health resources available at a family level. 

Because social media evolves rapidly, it is important to frequently examine the relationship these applications have with health and well-being. This particular study was unique in that it focused on parents’ use of social media and their self-reported family health. Families today are interacting with unprecedented technologies. Future research should involve longitudinal data to better understand the relationship between parent social media use and family well-being over time. Additional information could help parents become better consumers of social media and may also lead to improvements in app development to promote the health of its users. 

## Figures and Tables

**Table 1:** Demographics of Participants.

	Total Sample (*N* = 482)
Mother’s Age (mean/SD)	35.6 (7.05)
Father’s Age (mean/SD)	38.9 (8.15)
Married (percent)	90.04
White (percent)	74
Number of Children (mean/SD)	2.07 (0.95)
Income	
<$20,000 (percent)	4.15
>$100,000 (percent)	46.47
Mother’s Bachelor’s Degree or Higher (percent)	67.63
<High School Education (percent)	1.24
Father’s Bachelor’s Degree or Higher (percent)	67.84
<High School Education (percent)	2.9

**Table 2:** Means and standard deviations for social media application use among parents.

	Mother’s Social Media Use		Father’s Social Media Use	
	Mean	SD	Mean	SD
Facebook	5.16	1.42	4.92	1.42
YouTube	4.88	1.41	4.83	1.66
Instagram	4.38	1.92	4.1	2.06
Twitter	3.94	2.09	3.92	2.07
WhatsApp	3.79	2.23	3.84	2.25
Snapchat	3.66	2.04	3.35	2.04
Pinterest	3.45	1.85	3.05	1.92
Reddit	3.01	1.89	3.07	1.93
LinkedIn	3.01	1.9	3.08	1.92

Note: Likert scale response options for mean scores included 1 = never/almost never; 2 = every few weeks; 3 = 1–2 times/week; 4 = 3–5 times/week; 5 = daily; 6 = several times a day.

**Table 3:** Percentage of parents using social media, by reason.

**At Least One Application Primarily Used for Information**	
Mothers (percent)	62.66
Fathers (percent)	60.37
**At Least One Application Primarily Used for Entertainment**	
Mothers (percent)	93.57
Fathers (percent)	91.7
**At Least One Application Primarily Used for Connecting with Friends/Family**	
Mothers (percent)	78.84
Fathers (percent)	71.37

**Table 4:** Multiple linear regression results examining the associations of parental social media use and family health domains.

	Family Health Short-Form	Family Social and Emotional Health Processes	Family Healthy Lifestyle	Family Health Resources	Family External Social Supports
Mother’s Social Media Use Frequency	0.00	0.01	0.01 **	0.00	−0.01
Mother’s Use of Social Media to Obtain Information	0.01	0.05	0.01	−0.01	0.08
Mother’s Use of Social Media to Connect with Friends/Family	0.01	0.00	0.05	0.05	0.08
Mother’s Use of Social Media for Entertainment	0.00	0.04	−0.14	0.01	−0.04
Father’s Social Media Use Frequency	−0.00	−0.01	−0.00	−0.01	0.01
Father’s Use of Social Media to Obtain Information	0.02	0.05	0.04	−0.06	−0.06
Father’s Use of Social Media to Connect with Friends/Family	0.19 **	0.12	0.09	0.37 **	0.03
Father’s Use of Social Media for Entertainment	0.25 *	0.17	0.18	0.29	0.12

Note: Models control for mother’s age, race, education, and report of household income. * *p* < 0.05. ** *p* < 0.01.

## Data Availability

Those interested in the data may contact the corresponding author. Data are not publicly available per the approved Institutional Review Board proposal.
